# Outcomes of abdominoperineal resection for management of anal cancer in HIV-positive patients: a national case review

**DOI:** 10.1186/s12957-016-0970-x

**Published:** 2016-08-05

**Authors:** Ira L. Leeds, Hasan Alturki, Joseph K. Canner, Eric B. Schneider, Jonathan E. Efron, Elizabeth C. Wick, Susan L. Gearhart, Bashar Safar, Sandy H. Fang

**Affiliations:** 1 Department of Surgery, The Johns Hopkins Hospital, 600 North Wolfe Street, Blalock 618, Baltimore, MD 21287 USA; 2 Johns Hopkins Surgery Center for Outcomes Research, Department of Surgery, The Johns Hopkins Hospital, Baltimore, MD USA

**Keywords:** Anal cancer, Abdominoperineal resection, Human immunodeficiency virus infection, Surgical outcomes

## Abstract

**Background:**

The incidence of anal cancer in human immunodeficiency virus (HIV)-positive individuals is increasing, and how co-infection affects outcomes is not fully understood. This study sought to describe the current outcome disparities between anal cancer patients with and without HIV undergoing abdominoperineal resection (APR).

**Methods:**

A retrospective review of all US patients diagnosed with anal squamous cell carcinoma, undergoing an APR, was performed. Cases were identified using a weighted derivative of the Healthcare Utilization Project’s National Inpatient Sample (2000–2011). Patients greater than 60 years old were excluded after finding a skewed population distribution between those with and without HIV infection. Multivariable logistic regression and generalized linear modeling analysis examined factors associated with postoperative outcomes and cost. Perioperative complications, in-hospital mortality, length of hospital stay, and hospital costs were compared for those undergoing APR with and without HIV infection.

**Results:**

A total of 1725 patients diagnosed with anal squamous cell cancer undergoing APR were identified, of whom 308 (17.9 %) were HIV-positive. HIV-positive patients were younger than HIV-negative patients undergoing APR for anal cancer (median age 47 years old versus 51 years old, *p* < 0.001) and were more likely to be male (95.1 versus 30.6 %, *p* < 0.001). Postoperative hemorrhage was more frequent in the HIV-positive group (5.1 versus 1.5 %, *p* = 0.05). Mortality was low in both groups (0 % in HIV-positive versus 1.49 % in HIV-negative, *p* = 0.355), and length of stay (LOS) (10+ days; 75th percentile of patient data) was similar (36.9 % with HIV versus 29.8 % without HIV, *p* = 0.262).

Greater hospitalization costs were associated with patients who experienced a complication. However, there was no difference in hospitalization costs seen between HIV-positive and HIV-negative patients (*p* = 0.66).

**Conclusions:**

HIV status is not associated with worse postoperative recovery after APR for anal cancer as measured by length of stay or hospitalization cost. Further study may support APRs to be used more aggressively in HIV-positive patients with anal cancer.

**Electronic supplementary material:**

The online version of this article (doi:10.1186/s12957-016-0970-x) contains supplementary material, which is available to authorized users.

## Background

The incidence of squamous cell anal cancer in the last decade has been increasing [1], and the disease disproportionately affects HIV-positive individuals [2]. In non-elderly populations, anal cancer affects approximately 46 in 100,000 HIV-positive men and 10 in 100,000 HIV-positive women compared to 2 in 100,000 HIV-negative men and negligible rates for HIV-negative women [3]. Increasing evidence suggests that the HIV-positive population with anal cancer is distinctly different from the historical squamous cell anal cancer population [4]. Amongst HIV-positive individuals, the risk of anal cancer diagnosis is 25 times higher [2, 5], and HIV infection increases the risk of death from anal cancer by one to four times [4, 6, 7]. HIV-positive patients with anal cancer are more likely to die of anal cancer than those without HIV, and prior studies have suggested a less effective initial treatment response in the HIV-positive population to first-line anal cancer therapy [4, 8–12].

The National Comprehensive Cancer Network (NCCN) Clinical Practice Guidelines in Oncology for anal carcinoma recommends chemoradiation therapy as the primary treatment [13]. This recommendation arises from historical findings that the majority of patients who underwent surgical resection had a complete pathological response with no evidence of residual tumor in the pathological specimen after neoadjuvant chemoradiation therapy was administered. Chemoradiation rivals abdominoperineal resection in that it preserves anal sphincter function [14, 15]. For progressive and persistent disease, abdominoperineal resection is recommended.

Anal cancer is rare with less than 8000 reported cases in the USA annually [1, 16], and recurrent anal cancer eligible for salvage surgery is less than 5 % of total cases [17, 18]. All studies of recurrent squamous cell anal cancer that go beyond observational survival statistics are typically based on small case series usually reporting a single institution’s experience of only 100 or fewer cases [19, 20]. Moreover, the vast majority of these studies do not examine the relationship between HIV infection and anal cancer surgery outcomes even though the virus has been implicated in the rise of anal cancer and its worsening outcomes in recent years [1, 2, 4, 21]. Without larger case populations with good-quality data, the risk factors, trajectories, and subgroup disparities have been difficult to describe. This study aims to describe differences between anal cancer patients with and without HIV undergoing abdominoperineal resection (APR).

## Methods

### Data collection

Under Institutional Review Board approval, selection criteria for this study included all patients diagnosed with squamous cell anal cancer undergoing APRs from January 1, 2000 through December 31, 2011 using the Agency for Healthcare Research and Quality’s (AHRQ) Healthcare Utilization Project’s (HCUP) National Inpatient Sample (NIS). The NIS is the largest all-payer inpatient healthcare database in the USA and, with weighting, represents approximately 95 % (~20 % unweighted) of all hospitalized patients in the USA. Patients with anal cancer requiring an APR were identified by the International Classification of Diseases, 9th Revision (ICD-9) codes (anal cancer 154.2, 154.3; APR 48.5×), which were also used for comorbidity and complication reporting (Additional file 1: Table S1). ICD-9 coding was also used to group patients with (042) and without HIV as well as those with (86.7×) and without perineal closure tissue flaps.

### Study variables

Study variables were selected through a consensus-driven selection process using the available variables in the National Inpatient Sample and excluding those that five experienced colorectal surgeons found to be unlikely to be predictive of outcomes following abdominoperineal resection. Patient demographics were included in the study (e.g., age, sex, race) with age categorized into decades. Hospital characteristics (e.g., teaching vs. non-teaching hospital, urban vs. rural hospital) were included. Categories of comorbidities (e.g., diabetes; cardiovascular, liver, pulmonary, and renal) and postoperative complications (e.g., renal, cardiac, respiratory, liver, and gastrointestinal; deep venous thrombosis/pulmonary embolism, wound and intra-abdominal infections, sepsis and septic shock, postoperative hemorrhage, re-exploration) were identified using ICD-9 codes from external sources [22]. In-hospital mortality was examined. Using hospital-specific cost-to-charge ratios provided by HCUP, hospitalization costs per patient were derived. All dollar values were adjusted to 2011 equivalents.

### Statistical analysis

HCUP NIS data was obtained with weighting to the size of the US population. Continuous variables were compared using the Mann-Whitney test of medians. Categorical variables were compared using the chi-square test. Logistic regression models were used to examine which factors determined extended length of stay. A generalized linear model examined which factors increased hospitalization costs. A modified Park test was used to determine the appropriate distribution. Both models were built using forward and backward selection methods in which HIV status was forced into the model and variables were retained in the model if their *p* values were less than 0.20. A *p* value of <0.05 was considered statistically significant. All analyses were performed using Stata/MP® version 12.1 (StataCorp, College Station, TX).

## Results

Initially, a total of 3336 patients meeting selection criteria were identified. Further analysis demonstrated that the age distribution of HIV-negative patients with anal cancer was bimodal, with a substantial portion greater than age 60 while the HIV-positive population was narrowly concentrated in a range less than 60 years old with no HIV-positive patients with anal cancer greater than 60 years old. To control for age-related differences in both populations, HIV-negative patients greater than 60 years old (*n* = 1611) were excluded from further analysis. The demographics of the excluded subset are included in Table 1 for comparison.

**Table 1:** Patients' demographics and baseline characteristics

	HIV−	HIV+	*P*	HIV− >60 years
Total *N*	1417	308	–	1611
	*N* (%)	*N* (%)		*N* (%)
Patient characteristics				
Age (years)^a^				
Median (IQR)	51(46–56)	47(40–53)	<0.001	72(66–79)
<40	98 (6.9 %)	80 (25.9 %)	<0.001	
41–50	567 (40.0 %)	119 (38.5 %)		
51–60	752(53.0 %)	110 (35.6 %)		
60–70				711 (44.2 %)
>70				900 (55.9 %)
Gender^b^				
Male	432 (30.6 %)	293 (95.1 %)	<0.001	649 (44.1 %)
Female	981 (69.4 %)	15 (4.9 %)		956 (55.9 %)
Race^c^				
White	796 (77.9 %)	160 (60.6 %)	0.19	1020 (84.7 %)
Black	125 (12.3 %)	55 (20.7 %)		85 (7.0 %)
Hispanic	50 (4.9 %)	29 (10.8 %)		63 (5.2 %)
Asian	15 (1.5 %)	≤10(<4 %)		11 (0.8 %)
Native American	≤10 (<1 %)	0		0
Other	30 (2.9 %)	16 (6.0 %)		26 (2.1 %)
Comorbidities				
Diabetes	144 (10.1 %)	25 (8.2 %)	0.64	223 (13.8 %)
Cardiovascular	9 (0.6 %)	0	0.51	134 (8.3 %)
Liver	19 (1.3 %)	≤10 (3 %)	0.85	16 (1 %)
Pulmonary	180 (12.7 %)	30 (9.7 %)	0.49	299 (18.6 %)
Renal	20 (1.4 %)	≤10 (3 %)	0.95	43 (2.6 %)
AIDS	N/A	≤10 (3 %)		N/A
No comorbidity	1089 (76.8 %)	252 (81.8 %)		1055 (62.4 %)
Any 1 comorbidity	286 (20.2 %)	47 (15.2 %)		508 (31.6 %)
Any 2 comorbidities	43 (3.0 %)	9 (2.9 %)		88 (5.5 %)
Any 3 comorbidities	0	0		≤10 (0.6 %)
≥1 comorbidity	329 (23.2 %)	56 (18.1 %)	0.65	606 (37.6 %)
Perineal tissue flaps				
With	191 (13.5 %)	49 (16.0 %)	0.58	113 (7.0 %)
Without	1226 (86.5 %)	259 (84.0 %)		1497 (93.0 %)
Hospital type				
Teaching status^b^				
Teaching hospital	1067 (75.8 %)	239 (77.4 %)		929 (57.9 %)
Non-teaching hospital	340 (24.2 %)	70 (22.7 %)	0.80	676 (42.1 %)
Hospital location^b^				
Urban	1321 (93.8 %)	302 (98.1 %)	0.22	1469 (91.5 %)
Rural	87 (6.2 %)	≤10 (<3 %)		136 (8.48 %)

Small sample subsets that are non-zero but less than 11 patients are not reportable per HCUP NIS guidelines

^a^Median age is based on unweighted data

^b^Total numbers may not add up due to less than 1 % missing data

^c^25 % missing data on race (HIV− *N* = 1022; HIV+ *N* = 264; HIV− >60 *N* = 1204)

After excluding elderly patients, a total of 1725 patients diagnosed with anal squamous cell cancer undergoing APR were identified. Three hundred eight patients (17.9 %) were HIV-positive. HIV-positive patients were younger than HIV-negative patients undergoing APR for anal cancer with a median age of 47 versus 51 years (*p* < 0.001), and HIV-positive patients were also disproportionately male (95.1 versus 30.6 %, *p* < 0.001). There was no statistical difference in race, comorbidities (diabetes, cardiovascular, liver, pulmonary, renal), use of tissue flaps for perineal closure, whether the surgery was performed at a teaching hospital, and the type of community (urban versus rural). The characteristics of the two populations are presented in Table 1.

Postoperative APR outcomes were similar in both groups (Table 2). The in-hospital mortality rate was 0 % in HIV-positive versus 1.49 % in HIV-negative patients (*p* = 0.36), and the proportion of patients having an “extended LOS” (10 days or more based on 75th percentile of all patient stays) were similar (36.9 % with HIV versus 29.8 % without HIV, *p* = 0.26). The only significant difference in outcomes was that postoperative hemorrhage was more frequent in the HIV-positive group (5.1 versus 1.5 %, *p* = 0.05). There was no difference in the proportion of patients in each group developing any postoperative complications (renal, cardiac, respiratory, liver, gastrointestinal, venous thromboembolism, wound infections, and intra-abdominal infection, sepsis). The presence of one or more comorbidities increased the rate of postoperative complications, which also contributed to a longer hospital length of stay. However, there was no difference between HIV-positive and HIV-negative patients having at least one comorbidity (*p* = 0.65).

**Table 2:** Postoperative outcomes following APR for anal cancer, compared by HIV infection status

	HIV−	HIV+	*P*	HIV− >60
Total *N*	1417	308	–	1611
	*N* (%)	*N* (%)		*N* (%)
Postoperative complications				
Renal	106 (7.5 %)	28 (9.1 %)	0.66	154 (9.6 %)
Cardiac	≤10 (<1 %)	≤10 (<3 %)	0.38	68 (4.2 %)
Respiratory	221 (15.6 %)	56 (17.8 %)	0.67	258 (16.0 %)
Liver	0	0	0	0
Gastrointestinal	15 (1.0 %)	0	0.43	29 (1.8 %)
Venous thromboembolism	16 (1.2 %)	0	0.42	30 (1.8 %)
Wound infection and intra-abdominal infection	200 (14.1 %)	39 (12.5 %)	0.74	209 (13.0 %)
Sepsis	40 (2.8 %)	≤10 (<3 %)	0.40	71 (4.4 %)
Postoperative hemorrhage	19 (1.4 %)	16 (5.1 %)	0.05	46 (2.8 %)
Re-exploration	0	0	0	0
Any complication	447 (31.5 %)	107 (34.7 %)	0.62	613 (38.1 %)
In-hospital mortality	21 (1.5 %)	0 (0 %)	0.36	46 (2.9 %)
Length of stay^a^				
Median (IQR)	8(6–12)	9(7–14)	0.13	9(7–14)
Extended LOS	423(29.8 %)	114 (36.9 %)	0.26	644(40.0 %)
Cost^b^				
Median (IQR)	20,124 (13,529–29,264)	23,908 (15,915–34,378)	0.08	19,618 (13,822–23,757)
Mean (95 % CI)	26,588 (23,169–30,008)	29,899 (23,115–36,683)	0.39	27,948 (25,035–30,861)

Small sample subsets that are non-zero but less than 11 patients are not reportable per HCUP NIS guidelines

^a^Extended LOS: more than10 days (based on the 75th percentile of the data)

^b^Adjusted for 2011$. Total *N* for costs HIV− = 1212; HIV+ = 252; HIV− >60 = 1363

Multivariable regression was used to determine which factors significantly affected LOS and total hospitalization costs. Forward and backward selection for each model resulted in identical results. Regression analysis demonstrated no difference in extended LOS according to HIV status in the unadjusted (OR = 1.37, *p* = 0.26) and the adjusted (OR = 0.90, *p* = 0.79) model. However, male gender (OR = 1.85, *p* = 0.02), the occurrence of a postoperative complication (OR 5.00, *p* = 0.001), and the use of tissue flaps (OR 2.60, *p* = 0.008) were associated with an increased LOS (Table 3).

**Table 3:** Multivariable logistic regression model for extended length of stay after APR for anal cancer

Variables	Unadjusted OR (95 % CI)	*P* value	Adjusted OR (95 % CI)	*P* value
HIV	1.37 (0.78–2.41)	0.26	0.90 (0.43–1.91)	0.79
Any complication	5.09 (3.21–8.07)	<0.001	5 (3.11–7.98)	<0.001
Tissue flap	4.09 (2.11–7.95)	<0.001	2.60 (1.28–5.26)	0.008
Age (years)				
<40	Reference		Reference	
41–50	0.59 (0.29–1.21)	0.15	0.68 (0.31–1.49)	0.34
51–60	0.81 (0.41–1.63)	0.57	0.89 (0.40–1.91)	0.76
Sex				
Male	Reference		Reference	
Female	0.55 (0.36–0.84)	0.01	0.54 (0.33–0.91)	0.02

A generalized linear model was used to examine hospitalization costs. A modified Park test demonstrated that a gamma distribution was the best fit for hospitalization costs. The adjusted model showed that patients who experienced a complication, were treated at a teaching hospital, were closed with perineal tissue flaps, or had an extended length of stay also had greater hospitalization costs of $6914, $5662, $5008, and $21,162, respectively (all *p* < 0.001). However, there was no difference in hospitalization costs seen between HIV-positive and HIV-negative patients in both the unadjusted and the adjusted models (Table 4).

**Table 4:** Generalized linear modeling (gamma distribution) of total hospitalization costs after APR for anal cancer

Variables	Adjusted exp:coefficient^a^ (95 % CI)	Mean difference in cost (2011$)	*P* value
HIV	1.03 (0.86–1.22)	670	0.78
Any complication	1.29 (1.11–1.51)	6914	0.001
Tissue flap	1.20 (0.98–1.49)	5008	0.082
Age (years)			
<40	Reference	Reference	
41–50	1.03 (0.83–1.29)	859	0.76
51–60	1.12 (0.89–1.50)	2923	0.33
Sex			
Male	Reference	Reference	
Female	0.89 (0.77–1.03)	−3128	0.125
Teaching status			
Non-teaching	Reference	Reference	
Teaching	1.23 (1.08–1.41)	5662	<0.001
Extended LOS	2.20 (1.88–2.57)	21,162	<0.001

^a^Exponential coefficients

## Discussion

This study highlights important differences between those with and without HIV infection undergoing APR for anal cancer. In a weighted, national sample of patients requiring APR for anal cancer, HIV-positive patients were more likely to be male and, on average, younger than HIV-negative patients. However, this study also found important similarities between these two subpopulations with anal cancer requiring APR. Except for postoperative hemorrhage, which was more common in HIV-positive patients, there were no significant differences in complications, inpatient mortality, or length of stay between HIV-positive and HIV-negative patients. For median hospitalization costs, there was borderline significance; however, further analysis adjusting for other factors showed it to be both statistically insignificant.

Multivariable analysis demonstrated that the majority of variance in length of stay is explained by the occurrence of complications, more complex perineal closures, and the tendency for males to remain hospitalized longer than females, rather than a direct association with HIV infection. Gender differences were also found in hospitalization costs with females having lower costs than males. This trend may be driven by the larger number of males in the HIV-positive subset of anal cancer patients and the association of male gender with increased hospital length of stay following APR.

The limitations of our study deserve discussion. Many limitations of this study are inherent to the HCUP NIS database. First, this study was performed retrospectively using a national sample of patients. It is possible that surgeon selection bias of whom was selected for APR versus who was declined surgery may influence these results. The database does not differentiate outcomes based on surgeon experience or techniques for abdominoperineal resection. Long-term oncologic outcomes are also not followed through the NIS. Although the NIS is a sample of hospitalized patients from all US states and with appropriate stratified sampling represents 95 % of US inpatients, it still relies on hospital-reported ICD-9 codes for all the diagnoses and procedures studied here. The database contains administrative-level data and has the potential for coding errors and omissions. Hence, some components of patients’ care may not be recorded in the NIS [23].

The low prevalence of anal cancer requiring APR further limits this study’s conclusions. There are just over 7000 cases of anal cancer annually in the USA [1, 19], and less than 5 % of these cases require APR (e.g., late-stage disease, recurrent disease) [17, 18]. Furthermore, patients over the age of 60 were excluded to prevent confounding. The findings reported here are suggestive of no difference between patients undergoing APR for anal cancer with or without HIV infection, but the low prevalence limits statistical power. One important question for further exploration is whether the increased incidence of postoperative bleeding seen in HIV-positive patients—a relationship not previously seen—persists across other HIV-positive surgical patients, a potential unrecognized “idiopathic HIV-related functional coagulopathy”. However, it is important to note that this is the largest population of anal cancer patients undergoing APR ever studied, and there is no practical means to increase the size of the population studied in the near term.

Finally, this study is unable to assess long-term results regarding the post-hospital course of patients with anal cancer following APR. The NIS does not have any data following inpatient discharge, and the de-identified nature of the data prevents linking of NIS patient records to cancer databases that could potentially inform the matter. An important question this study is not able to answer is whether HIV infection status affects the long-term course of anal cancer after APR, such as rate of recurrence and overall survival.

Our hypothesis was that HIV-positive patients would do worse following an APR than HIV-negative patients due to recognized risk factors present in HIV-positive patients like atypical disease presentation, poor wound healing, and more expensive medical care [24–26]. However, our findings do not support this hypothesis. Our analysis suggests that HIV infection has no independent effect on perioperative mortality, length of stay, or hospitalization costs after accounting for other factors.

This conclusion is contrary to conventional wisdom. We believe there are two possible explanations for our findings. First, it is possible that the HIV-positive patients in this study were substantially healthier than historical populations previously studied. This study exclusively used data after January 1, 2000, while much of the previous literature examined HIV-associated disease at the height of the AIDS epidemic in an era prior to the introduction of highly active anti-retroviral therapy (HAART). Many studies specifically highlight low CD4 cell counts as a causal link to morbidity [24, 25]. It is also widely understood that CD4 cell counts rise with initiation of HAART [27]. One explanation for the lack of difference in outcomes between HIV-positive and HIV-negative anal cancer patients undergoing APR is that there may be little physiologic difference between these two populations. In the current era, it is possible that HIV-positive patients may no longer represent a unique population when it comes to surgical care if their HIV infection is well controlled on HAART. However, it is likely that there is considerable HIV-related variability within the population. For example, a 2012 study examined CD4 counts amongst HIV-positive patients undergoing chemotherapy for anal cancer and demonstrated median CD4 cell counts <300 suggesting ongoing active HIV infection [28]. Another limitation of the current study is the lack of CD4 counts to stratify patients by degree of immunosuppression.

An alternative explanation for the lack of significant difference between the two subsets studied has more concerning healthcare implications. Is it possible HIV-positive patients are thought to be too high-risk for high morbidity surgery like APRs and therefore being offered less optimal therapies instead? Previous studies have made the argument that APRs should be offered earlier to high-risk patients with anal cancer [29, 30]. One would expect HIV-positive patients to be disproportionately represented amongst those needing an APR and therefore should have worse outcomes. Perhaps the lack of difference in outcomes seen here is indicative of the need for liberalized selection of HIV-positive patients for APR to optimize long-term outcomes. Future prospective studies may need to account for the inherent selection of optimal surgical candidates from the broader population of those patients in which an APR for anal cancer is indicated.

## Conclusions

An 11-year retrospective study of the largest HIV population of patients undergoing APR for anal cancer shows that perioperative outcomes and costs are similar to HIV-negative patients undergoing APR. Further study of linked cancer datasets and prospective studies of HIV-positive patients diagnosed with anal cancer are necessary to better understand this phenomenon.

## Abbreviations

APR, abdominoperineal resection; HIV, human immunodeficiency virus

## Additional file

Additional file 1: Table S1. ICD-9 codes grouped by categories for patient comorbidities and in-hospital complications. (DOC 42 kb)

## References

[1] Nelson RA, Levine AM, Bernstein L, Smith DD, Lai LL (2013). Changing patterns of anal canal carcinoma in the United States. J Clin Oncol.

[2] Suneja G, Shiels MS, Angulo R, Copeland GE, Gonsalves L, Hakenewerth AM, Macomber KE, Melville SK, Engels EA (2014). Cancer treatment disparities in HIV-infected individuals in the United States. J Clin Oncol.

[3] Silverberg MJ, Lau B, Justice AC, Engels E, Gill MJ, Goedert JJ, Kirk GD, D'Souza G, Bosch RJ, Brooks JT (2012). Risk of anal cancer in HIV-infected and HIV-uninfected individuals in North America. Clin Infect Dis.

[4] Kim JH, Sarani B, Orkin BA, Young HA, White J, Tannebaum I, Stein S, Bennett B (2001). HIV-positive patients with anal carcinoma have poorer treatment tolerance and outcome than HIV-negative patients. Dis Colon Rectum.

[5] Grulich AE, van Leeuwen MT, Falster MO, Vajdic CM (2007). Incidence of cancers in people with HIV/AIDS compared with immunosuppressed transplant recipients: a meta-analysis. Lancet.

[6] American Cancer Society. Survival rates, by stage of anal cancer. Atlanta: American Cancer Society; 2014. http://www.cancer.org/cancer/analcancer/detailedguide/anal-cancer-survival-rates

[7] Biggar RJ, Engels EA, Ly S, Kahn A, Schymura MJ, Sackoff J, Virgo P, Pfeiffer RM (2005). Survival after cancer diagnosis in persons with AIDS. J Acquir Immune Defic Syndr.

[8] Oehler-Janne C, Huguet F, Provencher S, Seifert B, Negretti L, Riener MO, Bonet M, Allal AS, Ciernik IF (2008). HIV-specific differences in outcome of squamous cell carcinoma of the anal canal: a multicentric cohort study of HIV-positive patients receiving highly active antiretroviral therapy. J Clin Oncol.

[9] Meyer JE, Panico VJ, Marconato HM, Sherr DL, Christos P, Pirog EC (2013). HIV positivity but not HPV/p16 status is associated with higher recurrence rate in anal cancer. J Gastrointest Cancer.

[10] Goldstone SE, Johnstone AA, Moshier EL (2014). Long-term outcome of ablation of anal high-grade squamous intraepithelial lesions: recurrence and incidence of cancer. Dis Colon Rectum.

[11] Hogg ME, Popowich DA, Wang EC, Kiel KD, Stryker SJ, Halverson AL (2009). HIV and anal cancer outcomes: a single institution's experience. Dis Colon Rectum.

[12] Peddada AV, Smith DE, Rao AR, Frost DB, Kagan AR (1997). Chemotherapy and low-dose radiotherapy in the treatment of HIV-infected patients with carcinoma of the anal canal. Int J Radiat Oncol Biol Phys.

[13] Benson AB, Arnoletti JP, Bekaii-Saab T, Chan E, Chen YJ, Choti MA, Cooper HS, Dilawari RA, Engstrom PF, Enzinger PC (2012). Anal carcinoma, version 2.2012: featured updates to the NCCN guidelines. J Natl Compr Canc Netw.

[14] Nigro ND (1984). An evaluation of combined therapy for squamous cell cancer of the anal canal. Dis Colon Rectum.

[15] Nigro ND, Seydel HG, Considine B, Vaitkevicius VK, Leichman L, Kinzie JJ (1983). Combined preoperative radiation and chemotherapy for squamous cell carcinoma of the anal canal. Cancer.

[16] American Cancer Society: Cancer facts and figures 2014 [http://www.cancer.org/acs/groups/content/@research/documents/webcontent/acspc-042151.pdf].

[17] Kiran RP, Pokala N, Rottoli M, Fazio VW (2009). Is survival reduced for patients with anal cancer requiring surgery after failure of radiation? Analysis from a population study over two decades. Am Surg.

[18] Longo WE, Vernava AM, Wade TP, Coplin MA, Virgo KS, Johnson FE (1994). Recurrent squamous cell carcinoma of the anal canal. Predictors of initial treatment failure and results of salvage therapy. Ann Surg.

[19] Correa JH, Castro LS, Kesley R, Dias JA, Jesus JP, Olivatto LO, Martins IO, Lopasso FP (2013). Salvage abdominoperineal resection for anal cancer following chemoradiation: a proposed scoring system for predicting postoperative survival. J Surg Oncol.

[20] Lefevre JH, Corte H, Tiret E, Boccara D, Chaouat M, Touboul E, Svrcek M, Lefrancois M, Shields C, Parc Y (2012). Abdominoperineal resection for squamous cell anal carcinoma: survival and risk factors for recurrence. Ann Surg Oncol.

[21] Bilimoria KY, Bentrem DJ, Ko CY, Stewart AK, Winchester DP, Talamonti MS, Halverson AL (2008). Squamous cell carcinoma of the anal canal: utilization and outcomes of recommended treatment in the United States. Ann Surg Oncol.

[22] Dartmouth Atlas of Health Care. List of ICD-9-CM codes by chronic disease category. vol. 2015. Lebanon: Dartmouth Institute for Health Policy and Clinical Practice; 2008.

[23] Haut ER, Pronovost PJ, Schneider EB (2012). Limitations of administrative databases. JAMA.

[24] Leeds IL, Magee MJ, Kurbatova EV, del Rio C, Blumberg HM, Leonard MK, Kraft CS (2012). Site of extrapulmonary tuberculosis is associated with HIV infection. Clin Infect Dis.

[25] Lord RV (1997). Anorectal surgery in patients infected with human immunodeficiency virus: factors associated with delayed wound healing. Ann Surg.

[26] Schackman BR, Gebo KA, Walensky RP, Losina E, Muccio T, Sax PE, Weinstein MC, Seage GR, Moore RD, Freedberg KA (2006). The lifetime cost of current human immunodeficiency virus care in the United States. Med Care.

[27] Gibb DM, Newberry A, Klein N, de Rossi A, Grosch-Woerner I, Babiker A (2000). Immune repopulation after HAART in previously untreated HIV-1-infected children. Paediatric European Network for Treatment of AIDS (PENTA) Steering Committee. Lancet.

[28] Alfa-Wali M, Allen-Mersh T, Antoniou A, Tait D, Newsom-Davis T, Gazzard B, Nelson M, Bower M (2012). Chemoradiotherapy for anal cancer in HIV patients causes prolonged CD4 cell count suppression. Ann Oncol.

[29] Papaconstantinou HT, Bullard KM, Rothenberger DA, Madoff RD (2006). Salvage abdominoperineal resection after failed Nigro protocol: modest success, major morbidity. Colorectal Dis.

[30] Stewart D, Yan Y, Kodner IJ, Birnbaum E, Fleshman J, Myerson R, Dietz D (2007). Salvage surgery after failed chemoradiation for anal canal cancer: should the paradigm be changed for high-risk tumors?. J Gastrointest Surg.

