# Supplementary material for: Outcomes of abdominoperineal resection for management of anal cancer in HIV-positive patients: a national case review
Source: World J Surg Oncol. 2016 Aug 5;14:208. doi: 10.1186/s12957-016-0970-x (PMC4974747; doi:10.1186/s12957-016-0970-x)
Supplement: Additional file 1: Table S1. — ICD-9 codes grouped by categories for patient comorbidities and in-hospital complications. (DOC 42 kb) [file 12957_2016_970_MOESM1_ESM.doc]

**Table S1.** ICD-9 codes grouped by categories for patient comorbidities and in-hospital complications

| Variable | ICD-9 codes |
| --- | --- |
| Comorbidity | |
| Diabetes | 250, 250.1, 250.2, 250.4, 250.5, 250.6, 250.7, 250.8, 250.9 |
| Cardiovascular | 402.01, 402.11, 402.91, 404.01, 404.03, 404.11, 404.13, 404.91, 404.93, 428, 428.1, 428.2, 428.21, 428.22, 428.23, 428.3, 428.31, 428.32, 428.33, 428.,4 428.41, 428.42, 428.43, 428.9 |
| Pulmonary | 490, 491, 492, 494, 496, 493, 495 |
| Renal | 585, 585.1, 585.2, 585.3, 585.4, 585.5, 585.6, 585.9 |
| Liver | 571.2, 571.5, 571.6, 572.3 |
| Post-Operative Complications | |
| Renal | 584.5, 584.6, 584.7, 584.8, 584.9, 586, 590.10, 590.11, 595.0, 599.0, 997.5 |
| Cardiac | 410.00, 410.01, 410.10, 410.11, 410.20, 410.21, 410.30, 410.31, 410.40, 410.41, 410.50, 410.51, 410.60, 410.61, 410.70, 410.71, 410.80, 410.81, 410.90, 410.91, 410.02, 410.12, 410.20, 410.21, 410.22, 410.32, 410.42, 410.52, 410.62, 410.72, 410.91, 997.1 |
| Respiratory | 495.7, 507.0, 514, 512.1, 518.0, 518.4, 518.5, 518.81, 518.82, 519.02, 519.09, 519.1, 519.2, 997.3, 481, 482.0, 482.1, 482.2, 482.30, 482.31, 482.32, 482.39, 482.40, 482.41, 482.49, 482.81, 482.84, 482.89, 482.9, 483.8, 485, 486, 518.51, 518.52, 518.53, 518.84, 518.89, 482.42, 482.82, 482.83, 483.0, 483.1, 484.1, 484.3, 484.6, 484.5, 484.7, 484.8, 507.1, 507.8, |
| Liver | 570, 573.4, 572.0 |
| Gastrointestinal | 578.0, 578.1, 578.9, 530.10, 530.82, 531.00, 531.01, 531.11, 531.20, 531.21, 532.00, 532.01, 532.10, 532.11, 532.20, 532.21, 533.00, 533.01, 533.10, 533.11, 533.20, 533.21, 534.00, 534.01, 534.10, 534.11, 534.20, 534.21, 535.01, 535.11, 535.21, 535.41, 535.51, 535.61, 578.9 |
| Venous Thromboembolism | 453.81, 453.82, 453.83, 453.84, 453.85, 453.86, 453.87, 453.89, 453.40, 453.41, 453.42, 453.8, 453.9 |
| Wound Complication | 567.22, 567.21, 567.2, 567.23, 567.29, 567.8, 567.81, 567.82, 567.89, 567.9, 569.5, 590.2, 682, 682.2, 682.5, 730.00, 998.3, 998.30, 998.31, 998.32, 998.33, 998.5, 998.51, 998.59, 998.6, 998.83, |
| Sepsis | 038.0, 038.10, 038.11, 038.19, 038.3, 038.40, 038.9, 785.5, 785.50, 785.51, 785.52, 785.59, 790.7, 998.0 |
| Hemorrhage | 998.11, 998.12, 998.13, 998.51 |
| Re-exploration | 54.12, 54.61 |
